# An Analysis of the Structural Factors Affecting the Public Participation in Health Promotion

**DOI:** 10.5539/gjhs.v8n8p94

**Published:** 2015-12-17

**Authors:** Raheleh Ghaumi, Tayebe Aminee, Akram Aminaee, Mojgan Dastoury

**Affiliations:** 1 Research & Publication Institute of Peace Culture Charter, Tehran, Iran; 2 Unit of Adolecent and Youth Health and School in Sanitation Center of Golestan Province, Iran; 3 Research Institute of Koodakan Donya (World Children), Iran

**Keywords:** health promotion, public participation, CBPR

## Abstract

The present study focuses on analyzing national and international Community-Based Participatory Research (CBPR) studies published from 2000 to 2012 in order to identify and categorize the possible factors that affect social participation for improving the public health. Clearly, improving the public health necessitates a combination of the participation and responsibility by the social members and the attempts by public health policy-makers and planners. CBPR studies are selected as the corpus since they seek to encourage active and informed participation of the social members in fulfilling the health related goals. The present study is conducted through meta-synthesis within a qualitative framework. The results revealed a set of factors within the structural capacities which were employed by the CBPR researchers for achieving the health promotion goals. The structural capacities employed in the interventions could be considered on the cultural and social grounds. The cultural grounds were divided into scientific and religious attempts. For the scientific attempts, the results highlighted the participation of higher education institutes including universities and research centers as well as educational institutes such as schools and the relevant institutions. And regarding the religious attempts, the results indicated that the cooptation of religious centers played the greatest role in enhancing the public participation.

## 1. Introduction

### 1.1 Statement of the Problem

Today, the developed countries try to address health promotion at the same level as the disease prevention through the health care systems which might indicate to the increasing significance of this field. The key component of health promotion attempts is raising public awareness regarding effective factors involved in individual and social health and making appropriate decisions for a healthier lifestyle. Therefore, the theories, models and approaches introduced within the field of health promotion science mainly highlight the significance of interventions at the individual, group, organizational, and society levels and relevant policy making practices. Nevertheless, due to the significance of the behavioral and lifestyle factors, the sociological approach has gained more attention among the researchers and practitioners. This approach basically focuses on effective participation of the communities and their commitment for actions taken with the purpose of bringing about social and political changes.

The sociological approach introduces concepts such as healthy city, healthy village, healthy society, and health promoting schools as the fundamental concepts. In addition, it necessitates the immediate engagement of the governments in providing convenient health care services for different social classes and taking actions enhancing community members’ management and decision making skills for leading a healthier lifestyle. These could be considered as health promotion goals to be fulfilled, particularly for societies such as Iran where the health promotion field is dominated by the medical approach. Obtaining the defined goals in health promotion field requires, on the one hand, the commitment of the government for investing greater energy and funds in the relevant practices, and on the other hand, the awareness of the target individuals about how to intervene in public health promotion which could be enhanced through well-designed programs and attempts (Eslami, 2003).

The primary studies conducted within the health promotion field indicate to a theory - practice gap in social interventions and policy makings attempts. The Community Based Participation Research (CBPR) that seeks to enhance the effective public participation in the process of research as well as intervention might be considered as an attempt for filling this theory-practice gap. These studies, which include a wide range of participants from state and private sections as well as the society members, try to raise the awareness of these groups about of a specific issue or phenomenon with regard to the related social, cultural, and economical dynamics. These studies aim to bridge the gap between theoretical knowledge and the practices that the participants conduct (Evidence Report Technology Assessment, No. 99). In this way, CBPR could provide an efficient model for encouraging the public participation in health promotion practices and helping the authorities to identify the public needs and respond to them appropriately. For instance, *Essential National Health Research* (ENHR) is one of the attempts designed on the basis of social participation and mentions society as one of the three beneficiaries. Therefore, creating social changes is an important step toward making the studies more efficient and applicable (Robbie et al., 2008). Nevertheless, due to the lack of systematic knowledge the field public health promotion (Gutierrez & Lewis, 2005), we constantly face the question that which mechanisms could facilitate the effective and constructive participation of different social groups at the individual, organizational, and social levels.

Due to the culture-oriented nature of CBPR, the potential strengths and weaknesses of these studies might differ in various communities. Therefore, investigating and analyzing the contents of these studies could reveal the potential factors that improve the public participation in health promotion activities. Identifying the factors that affect the process of social involvement for the health promotion purposes could involve positive implications for the policy making attempts aimed at public welfare and health. Therefore, the present study aims to analyze the content of national (Iranian) and international CBPR studies in order to identify and categorize the factors that play a significant role in encouraging the public participation and intervention. This study seeks to classify the factors according to the priorities found in CBPR studies.

### 1.2 Theoretical Framework

Following the CBPR studies, some researchers have analyzed these studies in order to identify the effective factors, gaps, challenges, and the barriers for the application of the implications and comments provided by the CBPR. Malekafzali and his colleagues) 2010) argue that the freshness of this approach and lack of appropriate local patterns for attracting the public participation might be the possible reasons for lack of applicability of CBPR findings. This problem is partly related to the weakness of the structural capacities such as bureaucratic barriers (Holkup et al., 2004; Riley et al., 2009; Torrence & Guidry, 2007), democratic deficiencies, and lack of responsiveness by the institutions (Boyce, 2001). Clearly, the state sections play a significant role in social, cultural, and economic life of the society members; therefore, the support provided by the managers in service-executive sections is a leading factor in the success of the health promotion programs. Furthermore, the resilience of the state sections involved for responding to the cultural and social requirements and the complexities of the procedure of research, policy making, implementation processes are considered as crucial factors for strategic planning and managing these kinds of research (Riley et al., 2009).

Most of the CBPR studies highlight the important role of government in designing and conducting research programs, distributing resources, bridging the theory-practice gap, policy making, implementation phases, and encouraging inter-province (or inter-state) cooperation. These studies remind that the cooperation of the managers who could provide mutual support and share common goals are critical to the continuation of effective collaborative relationships to enhance efficiency of the interventions (Potvin et al., 2003, Riley et al., 2009). These factors could encourage and increase the participation of non-governmental health organizations or the charities in developing local/national policies and practices (Riley et al., 2009).

On the other hand, for strengthening the links between research, policy making, and implementation phases, that requires the committed cooperation by the research teams and translation of obtained knowledge into practices, we need to encourage the constructive relationship between universities, state research centers, and researchers. According to the studies in this field, taking the responsibility for these projects by the government could enhance the public trust and participation (Riley et al., 2009). The results of CBPR indicate to the lack of scientific and theoretical basis, and lack of systematic plans for implementing, evaluating, reporting, and publishing the results in several contexts (Riley et al., 2009; Dejman et al., 2012; Mohamadi, 2008). Furthermore, these factors would lead to failure in achieving acceptable levels of public participation (Flicker et al., 2008), difficulties in encouraging sustained public participation, and lack of public competencies and resources which might result from lack of public awareness raising regarding the health promotion issues (Sousa et al., 2010; Boyce, 2001).

According to the relevant studies, state institutions are capable of improving the public participation through the social capacities due to their potential power. Most of the state institutions such as schools and religious centers involve a variety of groups which shows that the cooperation of these institutions could facilitate the access to target population, particularly in under-developed societies where the civil organizations have not been developed as much as the developed societies. The religious centers, as one of the major institutions with great cultural acceptability, could maintain a great potential for enhancing public health practices and participation. These centers that are considered as cultural infrastructure could play a dynamic role in sustaining the health promotion educational programs. These centers, and the population which attend them which cover a range of civil, religious and political groups, could create considerable opportunities for identifying and prioritizing the health problems, and improving the public participation; particularly in religion-based programs (Torrence & Guidry, 2007; Wilcox et al., 2010).

CBPR programs could also intervene in improving children and adolescents’ health and participation through cooperating with schools. The acceptability and credibility of this educational institution among the public could pave the way for enhancing public participation in school-based health promotion programs (Lakes et al., 2009). In addition, the local policy making for schools need to focus on large-scale policy makings, identifying the barriers for participation of these age groups, and employing the effective techniques according to the cultural-social features of the target society in the schools (Goh et al., 2009). The cooperation of educational institutions with CBPR could lead to precious results such as improving self-regulation, citizenship, and responsiveness (Valaitis & Omara, 2005), and developing occupational skills through taking roles as managers, facilitators, or coordinators (Downey et al., 2010) that are determining factors in successful implementation of health promotion programs.

## 2. Methodology

### 2.1 Corpus

The corpus for this study included two sets of national (Persian) and international CBPR papers which were collected through a two-step procedure. In the first step, the papers and documents that focused on the public intervention and were published in the valid scientific data bases (in English and Persian) during the last ten years were selected through the relevant key words. Table 1 presents details considered for collecting the papers at the first step.

**Table 1:** The procedure for collecting the target corpus

The English and Persian key words	English: Community/Community-based/Social participation Participatory/CBPR/CBP intervention/Health promotion/Health promotion model/interventions/Initiation /initiative intervention/Participation
Where the keywords were occurred	Title/keywords/abstract
Time span	2000-2012
Data bases searched	Magiran/SID/IRANMEDEX/pubmed/CINAHL/All bases covered by the Digital Library of the Iran Ministry of Health and Medical Education (inlm)
Type of document	Paper/report/full-text document

In the first step, 139 English papers and 31 Persian papers were collected. In the second step, the papers which maintain one the following factors were included: 1. examined a health promotion intervention; 2. had a qualitative framework, a combination of qualitative and quantitative methods, or presented the qualitative data obtained from a study; 3. focused on investigating or identifying the effective factor(s) in encouraging the public participation; 4. focused on lessons learned from the studies on community-based health promotion. The papers that exclusively present theoretical discussions about social participation in public health were excluded from the corpus.

From the total 170 papers collected in the first step, 19 papers (18 English and 1 Persian) were selected as the final corpus. The selected papers were analyzed according to these criteria which were designed by researchers according to the previous studies (Zimmer, 2006; Xu, 2008; Finlayson & Dixon, 2008; Sandelowski & Barroso, 2003; Paterson et al., 2009): 1. The precision of the subject under study; 2. The coordination between method and the research design; 3. Clarity and considering the methodological and ethical principles; 4. Precision in data analysis phase; 5. Clarity of the findings and coordination between the findings and the research questions.

### 2.2 Data Analysis

In this part, the selected papers were precisely reviewed by the researchers and analyzed through coding approach in order to present the primary classifications. To this aim, the papers were first analyzed through logical-interpretational method and next they were divided into semantic units for the purpose of analysis. In this method, the concepts that highlighted the effective factors regarding the health promotion through social participation were coded through inducing the meaning and the obtained concepts were classified in the next step. In each study analyzed, a class of factors was introduced as the effective factors for encouraging the social participation in health promotion by the researchers (Table 2).

**Table 2:** The factors affecting public participation in health promotion according to CBPR studies from 2000 to 2012

Code	Author/year/country	The focus of study	Capacities involve in CBPR
A	Bonnie S. Ho, 2002/USA	Increasing the participation of families in the education of minority students in schools	-Participation of scientific and executive and service sections in governmental institutions; -Participation of community and NGOs in team research, considering socioeconomic status, accessibility of interventions for community, trust building through support of reference or trustworthy groups; considering ethics and cultural values, critical self-assessment, collective wisdom, community knowledge and skills, creating social motive and satisfaction;
B	Goh, ying-ying et al., 2009/USA	Decreasing obesity among the minority students in schools	-Participation of scientific, executive and service sections in governmental institutions; -Participation of community and NGOs in team research, considering socioeconomic status, accessibility of interventions for community, considering ethics and cultural values, critical self-assessment, collective wisdom and community knowledge and skills;
C	OttH, Carol, et al., 2003, USA	Assessing students’ personal and social development and health	-Participation of scientific, executive and service sections in governmental institutions; -Participation of community and NGOs in team research, considering socioeconomic status, accessibility of intervention for community, considering ethics and cultural values, critical self-assessment, collective wisdom and community knowledge and skills;
D	Riley L. B. et al., 2009/Canada	Reviewing the factors that facilitate and limit the intervention activities for public health	-Participation of executive and service sections in governmental institutions;
E	Potvin, Louise, et al., 2003/Canada	Preventing diabetes among the native Canadian students	-Participation of scientific, executive and service sections in governmental institutions; -Participation of community and NGOs in team research, considering socioeconomic status, accessibility of intervention for community, trust building through support of reference or trustworthy groups, considering ethics and cultural values, critical self-assessment, collective wisdom and community knowledge and skills, creating social motive and satisfaction;
F	Valaitis, Ruta; Lind, Omara. /2005 Canada	Encouraging students to participate in school-based social development activities	-Participation of scientific sections in governmental institutions; -Participation of community in team research, accessibility of intervention for community, trust building through support of reference or trustworthy groups, considering ethics and cultural values, critical self-assessment, collective wisdom and community knowledge and skills, creating social motive and satisfaction;
G	Maureen, R Benjamins;Steven, Whiyman, 2010/USA	Health promotion for Jewish religious minority students	-Participation of scientific, religious, executive and service sections in governmental institutions; -Participation of community and NGOs in team research, considering socioeconomic status, accessibility of intervention for community, trust building through support of reference or trustworthy groups, considering ethics and cultural values, critical self-assessment, collective wisdom and community knowledge and skills, creating social motive and satisfaction;
H	Downey H, Laura, et al. 2010/USA	Health promotion for rural population	-Participation of scientific, religious and executive and service sections in governmental institutions; -Participation of community in team research, considering socioeconomic status, accessibility of intervention for community, trust building through support of reference or trustworthy groups, considering ethics and cultural values, critical self-assessment, collective wisdom and community knowledge and skills, creating social motive and satisfaction;
I	Torrence, A. William; Jeffrey, J. Guidry, 2007/USA	Sexual health promotion for African-American students	-Participation of scientific, religious, and executive and service sections in governmental institutions; -Participation of community in team research, considering socioeconomic status, accessibility of intervention for community, trust building through support of reference or trustworthy groups, considering ethics and cultural values;
J	Holkup A, Patricia et al., 2004/USA	Health promotion for American-Indians	-Participation of scientific, service and executive sections in governmental institutions; -Participation of community in team research, considering socioeconomic status, trust building through support of reference or trustworthy groups, considering ethics and cultural values, critical self-assessment, collective wisdom and community knowledge and skills;
K	Lakes, D. Kimberly et al., 2009/USA	Health promotion for children with behavioral disorders in ethical minorities	-Participation of scientific, religious, executive and service sections in governmental institutions; -Participation of community in team research, considering socioeconomic status, accessibility of intervention for community, trust building through support of reference or trustworthy groups, considering ethics and cultural values, critical self-assessment, collective wisdom and community knowledge and skills, creating social motive and satisfaction;
L	GiShawn, A, Mane et al., 2010/USA	Health promotion among the African-American youths	-Participation of scientific, executive and service sections in governmental institutions; -Participation of community and NGOs in team research, considering socioeconomic status, accessibility of intervention for community, trust building through support of reference or trustworthy groups, considering ethics and cultural values, critical self-assessment, collective wisdom and community knowledge and skills, creating social motive and satisfaction;
M	Bates, Denise; Wiginton, L, Kristin, 2008/USA	Body, mental, and social health promotion in Hispanic regions	-Participation of scientific sections in governmental institutions; -Participation of NGOs in team research, considering socioeconomic status, critical self-assessment, collective wisdom and community knowledge and skills, creating social motive and satisfaction;
N	Parsai M.B et al. 2011/USA	Preventing high-risk behaviors among the ethical minorities and people from lower social and economic classes	-Participation of scientific, executive and service sections in governmental institutions; -Participation of community in team research, considering socioeconomic status, accessibility of intervention for community, trust building through support of reference or trustworthy groups, considering ethics and cultural values, critical self-assessment, collective wisdom and community knowledge and skills, creating social motive and satisfaction;
O	Wilcox, Sara et al., 2010/USA	Body and mental health promotion in terms of region among the African-Americans	-Participation of scientific, religious, executive and service sections in governmental institutions; -Participation of community in team research, considering socioeconomic status, accessibility of intervention for community, considering ethics and cultural values, critical self-assessment, collective wisdom and community knowledge and skills, creating social motive and satisfaction;
P	Olaseha O, I; M.K.C, Sridhar, 2006/Nigeria	Preventing infections in two cities from Nigeria	Participation of scientific, executive and service section in governmental institutions; -Participation of community and NGOs in team research, trust building through support of reference or trustworthy groups, considering ethics and cultural values, critical self-assessment and collective wisdom, creating social motive and satisfaction;
Q	Smith, Luara et al. 2010/USA	Promoting mental-social health among the ethical minorities	Participation of scientific, executive and service sections in governmental institutions; -Participation of community in team research, considering socioeconomic status, trust building through support of reference or trustworthy groups, considering ethics and cultural values, critical self-assessment, collective wisdom and community knowledge and skills, creating social motive and satisfaction;
R	M. Dejman et al., 2012 I.R Iran	Evaluating the strengths and weaknesses of CBPR studies in Iran	-Participation of scientific, executive and service sections in governmental institutions; -Participation of community and NGOs in team research, accessibility of interventions for community, considering ethics and cultural values; critical self-assessment, collective wisdom and community knowledge and skills, creating social motive and satisfaction;
S	B. Garbanati, Lourdes et al., 2005-2006/USA	Assessing the needs of Hispanic NGOs for preparation in crisis	-Participation of scientific and executive and service sections in governmental institutions; -Participation of NGOs, considering socioeconomic status, trust building through support of reference or trustworthy groups, considering ethics and cultural values, critical self-assessment, collective wisdom and community knowledge and skills, creating social motive and satisfaction;

After identifying and coding the effective factors indicated in CBPR studies, the potential relationships between the factors were analyzed through the following procedure:

1. *Reciprocal translation*: the findings that could be translated and turned into each other were selected from the papers analyzed;
2. *Refutational translation*: the inconsistent and contradictory key concepts and contents were coded in the papers;
3. *In-a-line-of-argument translation*: the factors within a concept range that were considered as parts of a whole were identified and coded in the papers.

Next the identified factors were listed and presented in a table (table 3) in order to facilitate understudying and concluding.

**Table 3:** Effective factors in improving the cooperation on structural grounds according to the analyzed papers

Capacity	Ground	Element	Institution	Strategic Activities	Frequency of activity	Total (share of ground)
1. Structural capacities	1. Cultural grounds	1. Scientific	1. Education institutions	1. Identifying the social/health needs	4	69 (64%)
				2. Managing and leading the research process scientifically	3	
				3. Providing the support/logistic services	9	
			2. Research and higher education institutions	1. Identifying the social/health needs	10	
				2. Managing and leading the research process scientifically	11	
				3. Providing the support/logistic services	5	
				4. Funding	1	
				5. Dissemination of data	18	
		2. Religious	1. Religious institutions	1. Identifying the social/health needs	1	
				2. Managing and leading the ideological projects religiously	3	
				3. Providing the support /logistic services	4	
	2. Social grounds	1. Executive and service	1. Executive and service institutions	1. Identifying the social/health needs	4	41 (36%)
				2. Managing and leading the projects	1	
				3. Providing support/logistic services	5	
				4. Funding	11	
				5. Organizing and facilitating the inter-sectional cooperation	13	
		2. Communication	1. Communication institution	1. The cooperation by mass media and public press	7	
Total						110 (100%)

### 2.3 Reliability and Validity of the Study

The procedure of data analysis in this study was reviewed and improved by an expert team. In addition, the study covers a transparent description of the steps taken throughout the project, including the data analysis phase, in order to approach the audit trial or confirmability criterion. In order to increase the validity of the data, the research team conducted constant inspection and observation simultaneously and describe the findings precisely.

## 3. Findings

The analysis of the data obtained in this study indicated that the CBPR researchers share relatively common definitions of public participation in health promotion practices. In 12 papers, the participants, as individuals or organizations, worked with the researchers and played the role of cooperators throughout different steps of the project including evaluation of the problem and the current situation, collecting data, planning and implementing the intervention, and reporting the results. These participants employed a variety of the personal skills they had in order to facilitate the process of research. The analysis indicated that in 2 studies the cooperators evaluated the attitudes, life experiences, and social-cultural knowledge of the subjects who participated in the intervention plan through pre-intervention needs assessment. Paper D offers no explanation about the manner of this cooperation; paper S investigated the solutions for maximizing the public cooperation through NGOs; and no intervention occurred in paper M due to the group and racial contradictions. In addition, the analysis showed that 7 studies used the public cooperation in the process of reporting the findings.

The structural capacities include the facilities, resources, and relationships which could be provided by the state agencies and institutions.

According to the results of this study, the factors identified under the structural capacities could be divided into two sub-categories: Cultural and social grounds. The cultural grounds cover the facilities, resources, and relationships within the cultural state and local institutions such as schools, universities. These grounds included two sets of elements: religious and scientific. The social grounds, as the other sub-category of the social capacities, include the facilities, resources, and relationships in different fields of social services provided by the state and formal. The social grounds include executive-service and communication elements. The executive-service elements refer to the institutions under the supervision of government ministries such as hospitals, counseling and psychological service centers, water and electricity official organizations, and the municipalities that are involved in the social welfare services. The communication element refers to the media and public press which broadcast the news and information (Table 3).

According to Table 3, the results showed that the educational institutions participated in indentifying the social needs in 4 cases, managing the research projects scientifically in 3 cases, and proving the support services in 9 cases. The research and higher education institutions participated in indentifying the social needs, managing the research projects scientifically, and providing the support services in 10, 11, and 15 cases, respectively. In addition, these institutions cooperated in funding the projects and reporting the results in 1 and 18 cases, respectively. The results also indicates that the religious institutions participated in identifying social needs, managing the projects scientifically, and providing support/logistic services in 1, 3, and 4 cases, respectively (Table 3).

Regarding the social grounds, the results indicated that the executive and service institutions cooperated in identifying social needs, providing support/logistic services, and funding in 4, 5, and 11 cases, respectively. And the mass Media and public press cooperated in 7 CBPR research projects among the 19 papers analyzed (Table 3).

Totally, the cultural grounds from the structural capacities which include schools, universities, research centers, and religious centers intervened in identifying social needs in 15 studies and cooperated in managing the projects scientifically in 17 studies. According to the obtained results, the executive and service institutions participated in the same activities in 4 and 1 cases, respectively. According to the results, the highest level of participation in investment and funding (11) was related to the executive and service institutions in structural capacities; however, the research and higher education institutions participated in 1 case for the same activity. Furthermore, in the projects which included samples from the around a country or a province as the statistical populations, the communication element from the structural capacities was responsible for broadcasting the results through the mass media and public press such as radio and television channels as well as the newspapers and the smaller units such as local media, declarations, and newsletters.

## 4. Conclusion

Addressing and eliminating the health promotion problems confronted by several communities require the constructive cooperation among three main institutions including the society, the NGOs and the governmental sections. These agents could work effectively at the local, national, and international levels in order to produce and exchange the scientific knowledge and skills, fundraising, technological progress, and policy making (Sheykhattari & Kamangar, 2010). The roles and significance determined for each of these institutions differ greatly according to the levels of general development in communities. However, the results of the relevant studies has developed a prevailing and growing consensus over the fact that the levels of public participation in developmental practices (including health promotion) in a society is clearly a function of cultural and economic resources available to the society members. Therefore, it could be said that the government has to allocate more time and funds to educating and encouraging the rural populations with the aim of achieving the developmental goals since these populations normally suffer from the lack of economic as well as information resources (Dooris & Heritage, 2009). In Other words, the government has to assume and play a more substantial role and develop and employ a variety of approaches and strategies in order to attract the public participation; particularly among the less-informed rural populations.

This study analyzed CBPR papers which focused on improving the public participation in public health promotion programs. The results indicated that the state and governmental institutions play a leading role in encouraging the public to participate in health-related programs. The attempts by these intuitions were mainly focused on cultural practices, particularly through the educational, higher education, and religious institutions. The results showed that higher education and research institutions were the most active institutions that contributed in CBPR projects substantially as the most important centers for producing knowledge. According to these results, most of the CBPR researchers considered social programs as cyclic and interactive processes and they highlighted the coordination between local communities and the researchers as a determining factor in bridging the theory-practice gap that exists in the field of public health promotion programs (Riley et al., 2009). Therefore, as the findings of this study revealed, the specialists, experts, and university graduates working at the scientific - cultural centers assumed the responsibilities for managing, leading, scientific evaluation, as well as reporting the findings. Furthermore, in most of papers the starting point of the intervention project was identifying the social/health problem by the relevant and expert institutions. Due to the significance of the scientific accuracy of methodology and theoretical framework in community-based studies, the contribution of the academic researchers as the scientific representatives could grantee the improvement of the public participation through directing the policy making and funding attempts toward the considerable health needs of the society (Riley et al., 2009).

The other cultural ground within the structural capacities was education whose substantial cooperation in the community-based studies could play a significant role in improving the physical and mental health of the society. The importance of children and adolescents’ health issues, the vulnerability of these groups, and the socially traumatic conditions that these groups might experience in state schools and among the peers (Goh, 2009), have encouraged several researchers to focus on this social group, because any attempt to improve this group could be considered as a valuable source for correcting the lifestyle and improving the future generation’s health (Downey et al., 2010; Holkup et al., 2009).

The religious centers and the relevant institutions, as another element of the cultural grounds in the structural capacities played a leading role in promoting the values, beliefs and lifestyles (Torrence et al., 2007; Wilcox et al., 2010). Although, the results of this study showed that these centers played a significant role in guiding and supervising the public in religious and ideological issues, they could facilitate improving the public participation by using their potential human resources and equipment through the social acceptance these institutions have among the community members. And with regard to the negative views and distrust that the CBPR participants showed toward the public health infrastructures, such as the bureaucratic and technocratic systems imposed on minorities or traditional societies, the religious instructions could take advantage of their social acceptance to meet health related goals. Considering this potential, several studies have focused on faith-centered programs to fight diseases including chronic cardiovascular diseases, cancer, and diabetes which have produced positive results (Torrence & Guidry, 2007; Wilcox et al., 2010).

The results of this study indicated that the executive and service, as another element of structural capacities, might either facilitate or limit the public participation in health promotion because it covers a range of effects on the social, cultural, and economical life of the community members. According to the results, some of the main factors that could foster the cooperation between executive and service institutions and the social studies for meeting the health promotion goals include the government’s flexibility, political commitment, and the supports provided by the managers in these institutions (Torrence et al., 2007; Holkup et al., 2004; Riley et al., 2009). In addition, some of the present state bureaucracies might limit the access and participation of the minorities to the health intervention that create barriers for the successful completion of intervention processes (Lakes et al., 2009). Therefore, removing the potential bureaucratic and obstacles could be considered an essential step toward improving the public participation. Furthermore, the governmental institutions could create the opportunities for the cooperation of non-governmental and social institutions in the health promotion projects and practices through maintaining the coherence among the relevant sections (Riley et al., 2009).

An overall review of the strategic actions taken within the structural capacities through the community-based interventions from 2000 to 2010 with the purpose of encouraging the social participation in the health promotion projects showed that the governmental sections had the greatest share in the cultural dimensions. In addition, the governmental sections played a significant role in the social dimension from the structural capacities through facilitating the inter-section relations (Figures 2 and 3). It should be mentioned that the present study was conducted within a qualitative framework through meta-synthesis; hence the results obtained might not be generalizable to other populations.

**Figure 1:**
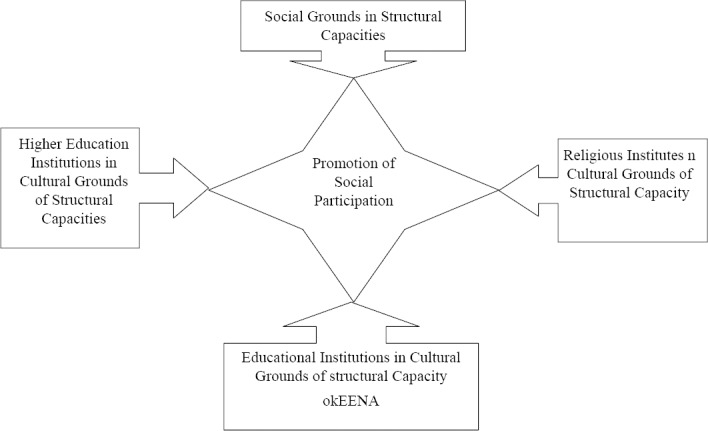
Diagram of structural and social capacities in the CBPR studies conducted from 2000 to 2010

**Figure 2:**
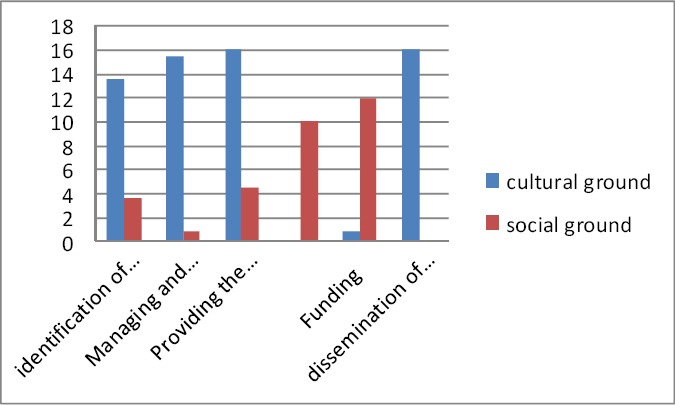
The frequencies of the strategic activities for increasing the social participation in CBPR interventions from 2000 to 2010 and the relevant dimensions in structural capacities

**Figure 3:**
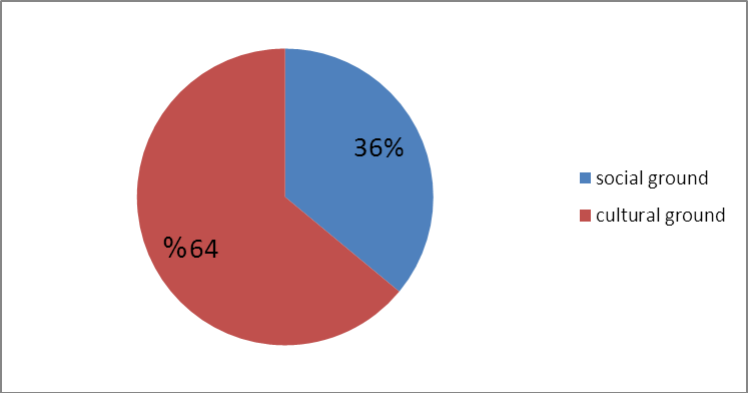
The share of each dimension in the structural capacities for improving the social participation in health promotion in CBPR studies conducted from 2000 to 2010
